# Graph Learning-Based Blockchain Phishing Account Detection with a Heterogeneous Transaction Graph

**DOI:** 10.3390/s23010463

**Published:** 2023-01-01

**Authors:** Jaehyeon Kim, Sejong Lee, Yushin Kim, Seyoung Ahn, Sunghyun Cho

**Affiliations:** 1 Department of Applied Artificial Intelligence, Major in Bio-Artificial Intelligence, Hanyang University, Ansan 15588, Republic of Korea; 2 Department of Computer Science and Engineering, Major in Bio-Artificial Intelligence, Hanyang University, Ansan 15588, Republic of Korea; 3 Department of Computer Science and Engineering, Hanyang University, Ansan 15588, Republic of Korea

**Keywords:** blockchain, cryptocurrency, phishing detection, graph learning, heterogeneous graph

## Abstract

Recently, cybercrimes that exploit the anonymity of blockchain are increasing. They steal blockchain users’ assets, threaten the network’s reliability, and destabilize the blockchain network. Therefore, it is necessary to detect blockchain cybercriminal accounts to protect users’ assets and sustain the blockchain ecosystem. Many studies have been conducted to detect cybercriminal accounts in the blockchain network. They represented blockchain transaction records as homogeneous transaction graphs that have a multi-edge. They also adopted graph learning algorithms to analyze transaction graphs. However, most graph learning algorithms are not efficient in multi-edge graphs, and homogeneous graphs ignore the heterogeneity of the blockchain network. In this paper, we propose a novel heterogeneous graph structure called an account-transaction graph, ATGraph. ATGraph represents a multi-edge as single edges by considering transactions as nodes. It allows graph learning more efficiently by eliminating multi-edges. Moreover, we compare the performance of ATGraph with homogeneous transaction graphs in various graph learning algorithms. The experimental results demonstrate that the detection performance using ATGraph as input outperforms that using homogeneous graphs as the input by up to 0.2 AUROC.

## 1. Introduction

Blockchain is a distributed ledger technology that facilitates the recording and managing of transactions in a decentralized manner. Participants in the blockchain network record and share ledgers by consensus. Ledgers are managed transparently without a central authority because all the participants share the same ledger. Owing to the advantages of blockchain technology, many fields such as finance, healthcare, and logistics have adopted blockchain technology [1,2,3]. In particular, cryptocurrencies such as Bitcoin [4] and Ethereum [5] are among the most successful blockchain applications in finance. Ethereum has introduced smart contracts that enable users to draft contracts without intermediaries to support various activities such as voting and auction [6]. With the introduction of smart contracts, Ethereum has become the most famous cryptocurrency platform. Users can transfer assets on cryptocurrency platforms without a central authority, such as a bank. Moreover, cryptocurrency platforms use pseudonymous transaction identities that allow users to trade assets without revealing their real-world identities.

However, many cybercrimes have been exploiting cryptocurrencies to hide their identities [7]. In 2017, WannaCry, one of the largest cybercrimes, encrypted victims’ data and demanded cryptocurrencies in exchange for the decryption key. WannaCry is estimated to have caused losses worth $4 billion and affected over 300k Windows computers in over 150 countries [8]. In Ethereum, smart contracts have created new opportunities for cybercrimes such as Ponzi schemes [9]. Chainanalysis, the blockchain data platform, reported that around 19,000 victims of Ethereum cybercrimes lost approximately $8000 per person only in 2017 [10]. Besides, the number of phishing accounts for more than 50% of all cybercrimes in Ethereum, indicating that phishing has become a critical issue in the Ethereum ecosystem [11]. The rise of phishing accounts on Ethereum has threatened users’ assets and the network’s reliability, destabilizing the Ethereum network [12]. Therefore, it is important to detect phishing accounts to protect users’ assets and make the Ethereum ecosystem more sustainable.

Blockchain transaction records are historical and publicly accessible data. Tracking and analyzing these records can infer the pattern of the specific accounts [13]. Many studies have been conducted to detect blockchain cybercriminal accounts by analyzing transaction records using machine learning [14,15]. Moreover, blockchain transaction records can be expressed in a graph structure with accounts as nodes and transactions as edges, such as typical financial transaction graphs. The graph structure of transaction records has motivated the exploration of graph-based learning approaches [16]. Based on this motivation, several studies have adopted graph-based learning methods such as network embedding algorithms and graph neural network (GNN) to detect cybercriminal accounts in the Ethereum network [17,18,19]. These studies adopted representing transactions as a homogeneous graph with a single type of node and a single type of edge. However, the homogeneous graph is difficult to describe for the character of the transaction records because it ignores the heterogeneity of the Ethereum network [20]. Moreover, the homogeneous transaction graph can have a multi-edge with multi-dimensional edge features between a node pair. Traditional graph learning approaches utilize one-dimensional edge features, which will limit learning the effectiveness of the multi-edge blockchain transaction graph [21,22].

In this paper, we propose a graph learning-based Ethereum phishing account detection framework with a heterogeneous transaction graph that has a multi-type node. First, we design a directed heterogeneous graph structure called an account-transaction graph (ATGraph), where transactions are considered nodes in the place of edges. ATGraph can effectively represent the Ethereum network’s heterogeneity. Moreover, it can reduce the complexity of Ethereum transaction graphs by representing multi-edge as single edges. According to the designed heterogeneous graph structure, we construct Ethereum transaction records into ATGraphs. Then, we extract features of nodes in ATGraphs based on their transactions. Finally, we perform graph learning-based supervised learning for graph classification to detect Ethereum phishing accounts. Our main contributions are summarized as follows:

### Contributions

- We propose a graph learning-based Ethereum phishing account detection framework with a heterogeneous transaction graph. Moreover, we design a novel directed heterogeneous graph structure called ATGraph to take the multi-edge into account and represent the heterogeneity of the Ethereum network.
- We conduct experiments on the Ethereum phishing account detection with various graph learning algorithms. In experiments, we compare the detection performance using ATGraphs and homogeneous graphs as inputs in each graph learning algorithm. Experimental results demonstrate that ATGraphs outperform homogeneous graphs in most graph learning algorithms.

The remainder of this paper is organized as follows. Section 2 introduces related works on the blockchain phishing account detection and graph learning approaches. Section 3 describes the methodology of the proposed Ethereum phishing account detection framework, including ATGraph construction and graph learning-based graph classification. In Section 4, we present the experimental results and analyses. Finally, Section 5 concluded this paper.

## 2. Related Work

### 2.1. Phishing Account Detection in Ethereum

The increase in phishing on Ethereum has become a major threat to the trading security of the Ethereum network [11]. Several studies have been conducted on phishing account detection to prevent cybercrimes in the Ethereum network.

In [14], Chen, W. et al. proposed a graph-based cascade feature extraction method and built the phishing scam identification model using a dual-sampling ensemble algorithm. The dual sampling ensemble method addresses a class imbalance problem by integrating the models trained by sampling examples and features. The results demonstrate that the dual sampling ensemble method represents graph features effectively.

The authors in [18] proposed a self-supervised incremental deep graph learning model for the phishing scam detection problem in the Ethereum network. It performed self-supervised and incremental learning using pretext tasks designed from spatial and temporal perspectives on the Ethereum transaction data. The results demonstrate that the proposed phishing scam detection outperforms the performance of other GNN models.

Wu, J. et al. [19] proposed a novel network embedding algorithm, trans2vec, to extract features of accounts for phishing identification. A one-class support vector machine classified the nodes into normal and phishing using the features extracted by trans2vec. The experimental results indicated that the trans2vec method outperformed other embedding methods in the graph data, such as Deepwalk [23] and Node2vec [24].

These studies represent transaction records as homogeneous graphs. However, the homogeneous transaction graph ignores the heterogeneity of the blockchain network. Moreover, it has a multi-edge between a node pair. Traditional graph learning algorithms have limitations in inefficiently learning graph structure that has multi-edge. Therefore, we need to consider a novel graph structure suitable for graph learning algorithms.

### 2.2. Graph Learning

Nowadays, there have been an increasing number of applications in which data are represented in the form of graphs. However, the complexity of graph data has imposed significant challenges on existing machine learning algorithms [25]. The main challenge of analyzing graphs with machine learning is that graph data do not exist in Euclidean space. It makes the interpretation of graph data more difficult than other Euclidean domain data, such as text, voice, and image.

Graph analysis algorithms, such as network embedding [26] and graph kernels [27], have been discussed to analyze non-Euclidean graph data. Network embedding methods aim to represent network nodes in low-dimensional vector representations. The graph kernel function measures the similarity between pairs of graphs such that kernel-based algorithms [28] can be used for supervised learning on graphs.

However, these algorithms suffer from computational bottlenecks and lose graph-level information representing the graph as a low-dimensional vector. GNN, which can be directly applied to graphs, was proposed to address these problems [29]. It learns the representation of a target node by iteratively propagating the neighbor information for the node, edge, or graph-level prediction. With the introduction of GNN, it is possible to extract high-level representations of graphs explicitly. Consequently, various graph analytic tasks such as classification, recommendation, and clustering can be effectively performed.

## 3. Proposed Framework for Ethereum Phishing Account Detection

**Figure 1:**
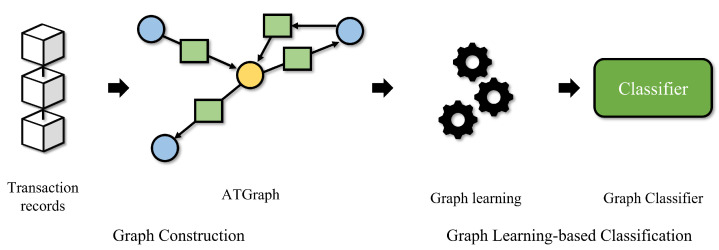
Overview of the proposed Ethereum phishing account detection framework.

In this section, we describe the proposed Ethereum phishing account detection framework. Figure 1 illustrates an overview of the proposed framework. The proposed framework comprises a graph construction and a graph learning-based classification phase. The graph construction phase generates ATGraphs and extracts node features by Ethereum transaction records. In the graph learning-based classification phase, graph learning algorithms and classifiers take ATGraph as input and detect Ethereum phishing accounts.

### 3.1. Graph Construction

We propose a novel heterogeneous graph structure called ATGraph to take multi-edge into account and represent the heterogeneity of the Ethereum network. In this section, we describe the definition, node feature extraction, and generation algorithm of ATGraph.

#### 3.1.1. Account-Transaction Graph

Ethereum transactions can be expressed as directed graph structures with accounts as nodes and transactions as edges. Most previous studies constructed transaction graphs as homogeneous graphs with single-type nodes. Homogeneous transaction graphs can have a multi-edge because multiple transactions can exist between a node pair. Multi-edge features are represented in the multi-dimensional feature matrix. However, most graph learning algorithms have not yet been demonstrated on the multi-edge because they utilize one-dimensional edge features for classification, ignoring rich edge features [21,22]. Moreover, homogeneous transaction graphs can not effectively represent the Ethereum network’s heterogeneity [20]. Therefore, we propose a novel heterogeneous transaction graph called ATGraph to take the multi-edge into account and the heterogeneity of the Ethereum network. ATGraph is a sub-graph of the Ethereum transaction graph, which contains a central node to be detected and its transactions. In the ATGraph structure, we consider transactions as nodes, not edges. In other words, ATGraph has two types of nodes: account nodes and transaction nodes. Multi-edges between a node pair can be expressed as single edges by expressing a transaction as a node. It represents multi-dimensional edge features as one-dimensional node features.

**Figure 2:**
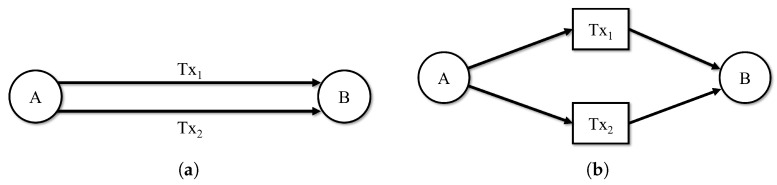
Graph structure of (**a**) the homogeneous transaction graph and (**b**) the proposed ATGraph.

Figure 2 shows structures of the homogeneous transaction graph and ATGraph. In these figures, there are two transactions $Tx_{1}$ and $Tx_{2}$ between node *A* and *B*. In Figure 2a, round nodes are account nodes and the edges are its transactions. The homogeneous transaction graph represents two transactions as multi-edge. In contrast, ATGraph expresses transactions as nodes, representing the multi-edge as single edges in Figure 2b, where round nodes are account nodes and square nodes are transaction nodes.

#### 3.1.2. Node Feature Extraction

For graph classification, the features of each node in the ATGraph are extracted. All features are calculated from Ethereum transaction records without any external data. The behavior pattern of the Ethereum account is represented by statistical, topological, and temporal features as listed in Table 1. The statistical features of the account consist of the total, minimum, maximum, and average values for the amount received and sent assets. The number of transactions directed into and out of the account is used as a topological feature. Temporal features include the lifetime of the account and time intervals between transactions. Each transaction contains the value and timestamp.

#### 3.1.3. ATGraph Generation

**Figure 3:**
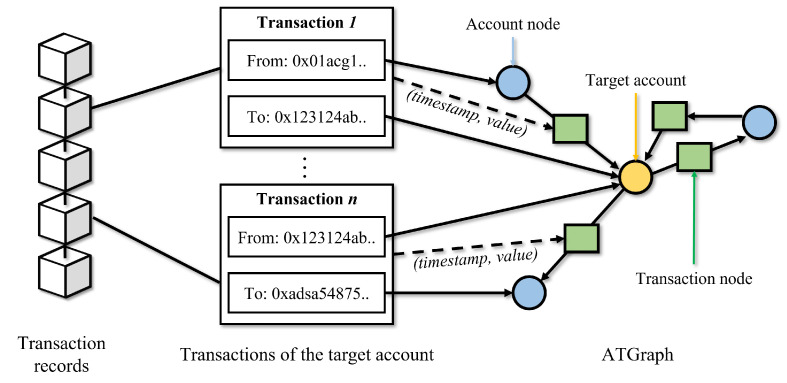
Overview of ATGraph generation based on Ethereum transaction records.

ATGraph can be generated based on the Ethereum transaction records as shown in Figure 3. Algorithm 1 describes the ATGraph construction algorithm. First, the algorithm initiates ATGraph $G_{a}=\left(V,E,r\right)$ and adds the target account node $v_{a}$ into the set of nodes V; *r* is labeled with the label of the target account *a* (rows 2–5). The transaction data *t* contains (i,j,timestamp,value), where account *i* transfers value to account *j* at timestamp. Based on the transaction data *t*, the account node $v_{i}$ or $v_{j}$ is added to V if it does not exist in V, and the transaction node $v_{t}$ is added to V (rows 7–12). Then, the edge $\left(v_{i},v_{t}\right)$ and $\left(v_{t},v_{j}\right)$ are added to the set of edges E (rows 13–15). (timestamp,value) are defined as transaction node features and are concatenated to the node feature matrix *X* (rows 16–17). After all nodes and edges are added to ATGraph $G_{a}$, the account node features are calculated and concatenated to the node feature matrix *X* (rows 19–22). As a result, the ATGraph generation algorithm returns ATGraph $G_{a}$ (row 23).
**Algorithm 1** ATGraph generation**Input:** The labeled Ethereum accounts *a*, The list of transactions $T_{a}$**Output:** The ATGraph $G_{a}$
 1:Feature matrix of nodes *X* 2:Initialize the directed graph $G_{a}=\left(V,E,r\right)$ 3:r← Label of *a* 4:$V\leftarrow \{v_{a}\}$ 5:E←{} 6:**for each** $t=\left(i,j,timestamp,value\right)\in T_{a}$ **do** 7:      **if** $v_{i}\notin V$ **then** 8:            $V\leftarrow V\cup \{v_{i}\}$ 9:      **end if**10:     **if** $v_{j}\notin V$ **then**11:            $V\leftarrow V\cup \{v_{j}\}$12:    **end if**13:    $V\leftarrow V\cup \{v_{t}\}$14:    $E\leftarrow E\cup \{\left(v_{i},v_{t}\right)\}$15:    $E\leftarrow E\cup \{\left(v_{t},v_{j}\right)\}$16:    $\vec{x_{t}}\leftarrow \left\{\left(timestamp,value\right)\right\}$17:    $X\leftarrow (X||\vec{x_{t}})$18:**end for**19:**for each** account node $v_{i}\in V$ **do**20:    $\vec{x_{i}}\leftarrow$ feature_extraction($v_{i}$)21:    $X\leftarrow (X||\vec{x_{i}})$22:**end for**23:**return** $G_{a}$


### 3.2. Graph Learning-Based Phishing Account Detection

This section describes the definition of the graph classification problem and the process of the graph classification method. We adopt supervised learning for graph classification to detect Ethereum phishing accounts. It takes ATGraph as input and returns the probability that the target account node is a phishing account.

#### 3.2.1. Problem Definition

The goal of the detection model is to classify the Ethereum account *a* by ATGraph $G_{a}$. $G_{a}=\left(V,E,r\right)$ is a directed heterogeneous graph, where each node v∈V can be Ethereum accounts consisting of *a* and its transactions $T_{a}$. $E=\{\left(v_{i},v_{t}\right),\left(v_{t},v_{j}\right)|v_{i},v_{t},v_{j}\in V\}$ is a set of edges, where $\left(v_{i},v_{t}\right)$ indicates the direction from account node $v_{i}$ to transaction node $v_{t}$ and vice versa. *r* is the label of *a*. In summary, the objective of the detection model can be expressed, that given a set of ATGraphs G, train a graph learning-based binary classifier to classify the types of *a*.

#### 3.2.2. Process of Graph Learning-Based Detection Method

**Figure 4:**
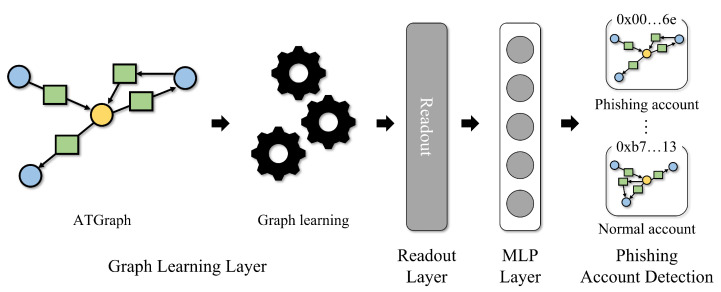
The overall processes of the graph learning-based Ethereum phishing account detection method.

The process of the proposed detection method consists of a graph learning layer, a readout layer, and a multi-layer perceptron (MLP) classifier. Figure 4 shows the process of graph learning-based Ethereum phishing account detection method. The graph learning layer generates a node representation of the input ATgraph. The readout operation generates a graph-level representation based on node representations. Finally, the MLP performs a binary classification for detecting phishing accounts. Graph learning-based graph classification for phishing account detection proceeds as follows.

(1) Graph Learning for Node-level Representations: Graph learning layers take ATGraph as inputs and learn node features using graph learning algorithms. Graph learning algorithms, such as network embedding and GNN models, can be used for node-level representation learning. Network embedding learns latent low-dimensional feature representations for the nodes and edges. Network embedding is to learn encodings for the nodes such that the similarity in the embedding space reflects the similarity in the network [30]. GNN adopted deep learning strategies, where input is fed into the hidden nodes to learn the representations and exploit relation information in the graph. GNN is to aggregate the features of neighboring nodes into the target node iteratively. After the graph learning process, graph learning layers return node-level representations of all nodes in the ATGraph.
(2) Readout for Graph-level Representation: The readout layer takes node-level representations and returns graph-level representations. The graph-level representation is a single vector representing the entire ATGraph. The readout layer obtains the graph-level representation by aggregating node-level representations by the mean or sum operation. We use the mean readout operation given as (1).
  (1) $$h_{G}=\frac{1}{N}\sum_{i=1}^{N}h_{i}^{\left(L\right)}$$
  where $h_{G}$ is the graph-level representation of ATGraph *G* and *N* is the number of units of node-level representation. $h^{\left(L\right)}$ is the node-level representations that are outputs of the graph learning layer.
(3) MLP for Graph Classification: The MLP classifier takes the graph-level representation $h_{G}$ as input and returns probabilities of account types. The MLP classifier classifies the category of *a* based on graph-level representations, which are the outputs of the readout layer. We use the cross-entropy loss function as the training objective given as (2).
  (2) $$L=-w_{y}log\frac{exp(x_{n,y})}{\sum_{i=1}^{C}exp\left(x_{n,c}\right)}$$
  where *x*, *y*, and *C* are the input, target, and number of classes, respectively.

Finally, the Ethereum phishing detection method classifies phishing accounts by the classification probability, which is the output of the MLP classifier.

## 4. Experiment and Results

This section describes experiments and results to evaluate the detection performance with ATGraphs. We compare the performance of ATGraph and homogeneous transaction graphs as inputs of various graph learning-based detection methods.

### 4.1. Data Collection

We collected labeled Ethereum accounts from authoritative sources for experiments listed in Table 2. We collected 1,659 verified phishing accounts and 1,700 normal accounts from XBlock (https://xblock.pro/) (accessed on 9 May 2022), Then, we crawled the transaction records of each labeled account using the API provided by *Etherscan* (https://etherscan.io/labelcloud/) (accessed on 9 May 2022). We collected 111,956 Ethereum accounts and 220,714 Ethereum transactions by parsing these transaction records.

### 4.2. Dataset and Evaluation Metrics

We constructed 3359 ATGraphs and homogeneous transaction graphs for each labeled Ethereum account. In the case of homogeneous transaction graphs, edge features are set to transaction node features of ATGraph. ATGraphs include 332,670 nodes and 441,428 edges and homogeneous transaction graphs include 111,956 nodes and 220,714 edges. We adopted five metrics to evaluate the performance of the ATGraph including accuracy, precision, recall, the F1 score, and the area under the receiver operating characteristic (AUROC). The precision indicates the number of accurate predictions from the perspective of the prediction results. The recall indicates the number of true positive classes that are successfully recalled. The F1 score is the harmonic mean of the precision and recall used for the imbalanced dataset. The AUROC indicates the model’s ability to discriminate between positive and negative examples. Note that higher accuracy, precision, recall, F1 score, and AUROC indicate better prediction performance.

### 4.3. Experimental Setup

In the experiments, we adopted four graph learning algorithms to evaluate the performance of ATGraph including SF [31], Graph2Vec [32], graph convolutional network (GCN) [33], graph isomorphism network (GIN) [34], and GraphSAGE [35]. SF and Graph2Vec are the network embedding algorithm, and others are the GNN models. We set the parameters of Graph2Vec as the default provided by the library with *wl_iterations* = 2, *dimensions* = 128, *down_samplings* = 0.0001, and *min_count* = 5. For SF, we set the parameters as a default with *dimension* = 128 and *seed* = 42. We used Adam [36] as the optimization method in GNN models. We set the parameters of GNN models with *n_layers* = {2, 3, 4}, *batch_size* = 64, and *hidden_unit* = {16, 32, 64, 128}. In the GIN, we used sum, max, and mean aggregators. For the GraphSAGE, we used mean, GCN, and pool aggregators. The candidates of the parameters are shown as Table 3. We found a set of parameters for the best performance using the grid search with 5-fold cross-validation in all approaches, including homogeneous transaction graphs and ATGraph. The models were run in PyTorch 1.11 [37]. We used a deep graph library (DGL) 0.8.0 [38] for the implementation of GNN models. For graph embedding algorithms, we used a karateclub library 1.2.3 [39] and NetworkX 2.5.1 [40].

### 4.4. Experimental Results and Analysis

#### 4.4.1. Performance Comparison Analysis

We compare the performance of phishing account detection using ATGraph and the homogeneous transaction graph as input to evaluate ATGraph. The results of phishing account detection performance with the best parameters are presented in Table 4. The performance of phishing account detection with ATGraph outperforms the performance with homogeneous transaction graphs in all graph learning algorithms. SF and Graph2Vec show the biggest performance difference of more than about 0.2 AUROC. It is because these methods are a network embedding algorithm based on a lower-dimensional structure. The network embedding algorithm utilizes the similarity of neighbor nodes, which means that it has a limitation in embedding multi-dimension edge features. In contrast, GraphSAGE has the lowest performance gain between the ATGraph and the homogeneous transaction graph. GraphSAGE aggregates neighbor node features and edge features to generate the center node features. It indicates that the detection performance with ATGraphs is more efficient in graph learning methods that utilize edge features less, such as SF. Moreover, the average AUROC with homogeneous transaction graphs is 0.8110, and that with the ATGraph is 0.9085. ATGraph outperforms the homogeneous graph by 0.0975 AUROC. It indicates that ATGraph eliminates multi-edges, making graph learning algorithms learn graph structures more efficiently Consequently, the detection performance using ATGraph as input outperforms using homogeneous transaction graphs, especially in the network embedding algorithms.

#### 4.4.2. Node Features Analysis

We compared node features between normal and phishing accounts to provide a better understanding of cybercriminal accounts. Table 5 compares normal and phishing account nodes’ average value of features. There are several notable feature differences. Phishing accounts have a short lifetime than normal accounts. Normal users use the same account to transfer their assets for a long period. In contrast, phishing accounts have a short active time versus normal accounts because they only used these accounts for fraud in a short period. Moreover, the balance of phishing accounts almost converges to zero. It is because phishing accounts are only used for fraud and transfer assets to their actual activated accounts. Therefore, the balance of normal accounts is more than zero, whereas the balance of phishing accounts is almost zero. For this reason, the ratio between the average of in-value and out-value also has a notable difference. Normal accounts have more in-value than out-value, but phishing accounts have more out-value than in-value. One more notable feature difference is the average interval between transactions. Phishing accounts steal multiple users’ assets in a short period because they are active for a short period. It means that there are many transactions for a short period in the phishing account. Therefore, the average interval between transactions of phishing accounts is shorter than in normal accounts.

## 5. Conclusions

In this paper, we proposed graph learning-based blockchain phishing account detection with a heterogeneous transaction graph. We proposed a novel graph structure called ATGraph, which represents transactions as nodes to eliminate the multi-edge. With ATGraph as input, graph learning algorithms can embed a graph structure effectively. We conducted experiments comparing Ethereum phishing account detection performance between ATGraph and homogeneous transaction graphs. The experimental results demonstrate that the detection performance with ATGraph outperforms that of a homogeneous transaction graph by up to 0.2 AUROC. It indicates that detecting phishing accounts with ATGraph is more efficient than using homogeneous transaction graphs as input. Moreover, ATGraphs are more efficient in the network embedding algorithms, which do not utilize edge features. We expect cybercriminal account detection to make blockchain networks more sustainable by regulating or blacklisting these accounts.

This work has several issues to be improved. ATGraph has a trade-off in the size of the graph. The number of nodes and edges is more than in homogeneous transaction graphs because ATGraph represents transaction edges as transaction nodes. Therefore, the more nodes and edges, the more time consumed to learn ATGraph than homogeneous transaction graphs. Moreover, the Ethereum network has two types of accounts, such as contract accounts and externally owned accounts, but we did not consider these account types in this work. In the future, we will design a novel graph learning model suitable for ATGraph to detect phishing accounts more effectively. In addition, we will consider various node/edge types to represent the heterogeneity of the Ethereum network more.

## Figures and Tables

**Table 1:** The description of node features.

Node Type	Feature	Description
Account node	In-degree	The number of received transactions.
	Out-degree	The number of sent transactions.
	In-value	The sum of the received value.
	Out-value	The sum of the sent value.
	Average in-value	The average of the received value.
	Average out-value	The average of the sent value.
	Min in-value	The minimum received value.
	Min out-value	The minimum sent value.
	Max in-value	The maximum received value.
	Max out-value	The maximum sent value.
	Lifetime	The active time of the account.
	Balance	The balance over the lifetime of the account.
	Average Inter-Tx Time	The average time interval between transactions.
Transaction node	Timestamp	Timestamp when a transaction was issued.
	Value	Amount of value in the transaction.

**Table 2:** Summary of collected data.

Data	Number of Data	Source
Normal account	1659	Xblock
Phishing account	1700	Xblock
Unlabeled account	108,597	Etherscan
Transaction	220,714	Etherscan

**Table 3:** The candidates of the parameters. The best parameters based on the AUROC are indicated in bold.

Algorithms	Parameters	Value
SF	dimensions	**128**
	seed	**42**
Graph2Vec	wl_iterations	**2**
	dimensions	**128**
	down_samplings	**0.0001**
	min_count	**5**
GCN	n_layers	2, 3, **4**
	batch_size	**64**
	hidden_unit	16, 32, 64, **128**
GIN	n_layers	2, 3, **4**
	batch_size	**64**
	hidden_unit	16, 32, 64, **128**
	aggregator	sum, **max**, mean
GraphSAGE	n_layers	2, 3, **4**
	batch_size	**64**
	hidden_unit	16, 32, **64**, 128
	aggregator	mean, GCN, **pool**

**Table 4:** Performance comparison using ATGraph and homogeneous transaction graph as input.

Input Graph	Method	Accuracy	Precision	Recall	F1-Score	AUROC
Homogeneous transaction graph	SF	0.5818	0.5804	0.5818	0.5805	0.5777
	Graph2Vec	0.6342	0.6337	0.6343	0.6342	0.6323
	GCN	0.9086	0.9082	0.9172	0.9086	0.9097
	GIN	0.9494	0.9494	0.9519	0.9494	0.9500
	GraphSAGE	0.9851	0.9851	0.9854	0.9851	0.9852
Average		0.8118	0.8114	0.8141	0.8116	0.8110
ATGraph	SF	0.7738	0.7822	0.7738	0.7737	0.7779
	Graph2Vec	0.8143	0.8144	0.8158	0.8143	0.8150
	GCN	0.9816	0.9816	0.9817	0.9816	0.9816
	GIN	0.9804	0.9803	0.9809	0.9804	0.9802
	GraphSAGE	0.9878	0.9878	0.9880	0.9878	0.9876
Average		0.9076	0.9093	0.9080	0.9076	0.9085

**Table 5:** The average value of account node features. The result shows that phishing accounts have the most notable differences in temporal features such as the lifetime and the average interval of transactions indicated in bold.

Features	Normal Account	Phishing Account
In-degree	76	23
Out-degree	23	8
In-value	18,676	138
Out-value	14,157	162
Average in-value	1922	40
Average out-value	1045	45
Min in-value	580	27
Min out-value	217	28
Max in-value	6056	90
Max out-value	4189	120
**Lifetime**	134,158,600	**75,671,864**
Balance	4519	−23
**Average Inter-Tx Time**	788,229	**541,912**

## Data Availability

The datasets generated during the current study are available from the authors on reasonable request.

## Abbreviations

The following abbreviations are used in this manuscript:

ATGraph	Account-transaction graph
AUROC	Area under the receiver operating characteristic
GCN	Graph convolutional network
GIN	Graph isomorphism network
GNN	Graph neural network
MLP	Multi-layer perceptron

## Notations

The following notations are used in this manuscript:

*a*	The labeled Ethereum account
S	Set of labeled Ethereum accounts
$T_{a}$	The set of account *a*’s transactions
$G_{a}$	The ATGraph of account *a*
G	Set of ATGraphs
V	Set of nodes in the graph
E	Set of edges in the graph
*r*	The label of account *a* in the graph
$\vec{x_{i}}$	Feature vector of the node $v_{i}$
*X*	Feature matrix of nodes
(p||q)	The concatenation of *p* and *q*

## References

[1] Patel S.B., Bhattacharya P., Tanwar S., Kumar N. (2020). Kirti: A blockchain-based credit recommender system for financial institutions. IEEE Trans. Netw. Sci. Eng..

[2] Bhattacharya P., Tanwar S., Bodkhe U., Tyagi S., Kumar N. (2019). Bindaas: Blockchain-based deep-learning as-a-service in healthcare 4.0 applications. IEEE Trans. Netw. Sci. Eng..

[3] Pournader M., Shi Y., Seuring S., Koh S.L. (2020). Blockchain applications in supply chains, transport and logistics: A systematic review of the literature. Int. J. Prod. Res..

[4] Nakamoto S. (2008). Bitcoin: A peer-to-peer electronic cash system. Decentralized Bus. Rev..

[5] Buterin V. (2014). A Next-Generation Smart Contract and Decentralized Application Platform. https://github.com/ethereum/wiki/wiki/White-Paper.

[6] Mohanta B.K., Panda S.S., Jena D. An overview of smart contract and use cases in blockchain technology. Proceedings of the 2018 9th International Conference on Computing, Communication and Networking Technologies (ICCCNT).

[7] Reddy E., Minnaar A. (2018). Cryptocurrency: A tool and target for cybercrime. Acta Criminol. Afr. J. Criminol. Vict..

[8] Chen Q., Bridges R.A. Automated behavioral analysis of malware: A case study of wannacry ransomware. Proceedings of the 2017 16th IEEE International Conference on Machine Learning and Applications (ICMLA).

[9] Bartoletti M., Carta S., Cimoli T., Saia R. (2020). Dissecting Ponzi schemes on Ethereum: Identification, analysis, and impact. Future Gener. Comput. Syst..

[10] Team A.C. (2017). The Rise of Cybercrime on Ethereum. https://blog.chainalysis.com/reports/the-rise-of-cybercrime-on-ethereum/.

[11] Conti M., Kumar E.S., Lal C., Ruj S. (2018). A survey on security and privacy issues of bitcoin. IEEE Commun. Surv. Tutor..

[12] Corbet S., Cumming D.J., Lucey B.M., Peat M., Vigne S.A. (2020). The destabilising effects of cryptocurrency cybercriminality. Econ. Lett..

[13] Kim J., Lee S., Kim Y., Cho S. A Graph Embedding-based Identity Inference Attack on Blockchain Systems. Proceedings of the 2022 International Conference on Electronics, Information, and Communication (ICEIC).

[14] Chen W., Guo X., Chen Z., Zheng Z., Lu Y. Phishing Scam Detection on Ethereum: Towards Financial Security for Blockchain Ecosystem. Proceedings of the Twenty-Ninth International Joint Conference on Artificial Intelligence.

[15] Ostapowicz M., Żbikowski K. (2020). Detecting fraudulent accounts on blockchain: A supervised approach. Proceedings of the International Conference on Web Information Systems Engineering.

[16] Zhou J., Cui G., Hu S., Zhang Z., Yang C., Liu Z., Wang L., Li C., Sun M. (2020). Graph neural networks: A review of methods and applications. AI Open.

[17] Yuan Z., Yuan Q., Wu J. (2020). Phishing detection on Ethereum via learning representation of transaction subgraphs. Proceedings of the International Conference on Blockchain and Trustworthy Systems.

[18] Li S., Xu F., Wang R., Zhong S. (2021). Self-supervised Incremental Deep Graph Learning for Ethereum Phishing Scam Detection. arXiv.

[19] Wu J., Yuan Q., Lin D., You W., Chen W., Chen C., Zheng Z. (2020). Who are the phishers? phishing scam detection on ethereum via network embedding. IEEE Trans. Syst. Man Cybern. Syst..

[20] Wang Y., Liu Z., Xu J., Yan W. (2022). Heterogeneous Network Representation Learning Approach for Ethereum Identity Identification. IEEE Trans. Comput. Soc. Syst..

[21] Xiong C., Li W., Liu Y., Wang M. (2021). Multi-Dimensional Edge Features Graph Neural Network on Few-Shot Image Classification. IEEE Signal Process. Lett..

[22] Gong L., Cheng Q. Exploiting Edge Features for Graph Neural Networks. Proceedings of the 2019 IEEE/CVF Conference on Computer Vision and Pattern Recognition (CVPR).

[23] Perozzi B., Al-Rfou R., Skiena S. (2014). DeepWalk: Online Learning of Social Representations. Proceedings of the 20th ACM SIGKDD International Conference on Knowledge Discovery and Data Mining (KDD ’14).

[24] Grover A., Leskovec J. (2016). Node2vec: Scalable Feature Learning for Networks. Proceedings of the 22nd ACM SIGKDD International Conference on Knowledge Discovery and Data Mining (KDD ’16).

[25] Wu Z., Pan S., Chen F., Long G., Zhang C., Yu P.S. (2021). A Comprehensive Survey on Graph Neural Networks. IEEE Trans. Neural Netw. Learn. Syst..

[26] Hamilton W.L., Ying R., Leskovec J. (2017). Representation learning on graphs: Methods and applications. arXiv.

[27] Vishwanathan S.V.N., Schraudolph N.N., Kondor R., Borgwardt K.M. (2010). Graph kernels. J. Mach. Learn. Res..

[28] Hofmann T., Schölkopf B., Smola A.J. (2008). Kernel methods in machine learning. Ann. Stat..

[29] Gori M., Monfardini G., Scarselli F. A new model for learning in graph domains. Proceedings of the 2005 IEEE International Joint Conference on Neural Networks.

[30] Arsov N., Mirceva G. (2019). Network embedding: An overview. arXiv.

[31] De Lara N., Pineau E. (2018). A Simple Baseline Algorithm for Graph Classification. arXiv.

[32] Narayanan A., Chandramohan M., Venkatesan R., Chen L., Liu Y., Jaiswal S. (2017). graph2vec: Learning Distributed Representations of Graphs. arXiv.

[33] Kipf T.N., Welling M. (2016). Semi-Supervised Classification with Graph Convolutional Networks. arXiv.

[34] Xu K., Hu W., Leskovec J., Jegelka S. (2018). How Powerful are Graph Neural Networks?. arXiv.

[35] Hamilton W., Ying Z., Leskovec J., Guyon I., Luxburg U.V., Bengio S., Wallach H., Fergus R., Vishwanathan S., Garnett R. (2017). Inductive Representation Learning on Large Graphs. Proceedings of the Advances in Neural Information Processing Systems.

[36] Kingma D.P., Ba J. (2014). Adam: A Method for Stochastic Optimization. arXiv.

[37] Paszke A., Gross S., Massa F., Lerer A., Bradbury J., Chanan G., Killeen T., Lin Z., Gimelshein N., Antiga L. (2019). Pytorch: An imperative style, high-performance deep learning library. Adv. Neural Inf. Process. Syst..

[38] Wang M., Zheng D., Ye Z., Gan Q., Li M., Song X., Zhou J., Ma C., Yu L., Gai Y. (2019). Deep graph library: A graph-centric, highly-performant package for graph neural networks. arXiv.

[39] Rozemberczki B., Kiss O., Sarkar R. (2020). Karate Club: An API Oriented Open-source Python Framework for Unsupervised Learning on Graphs. Proceedings of the 29th ACM International Conference on Information and Knowledge Management (CIKM ’20).

[40] Hagberg A., Swart P., S Chult D. (2008). Exploring Network Structure, Dynamics, and Function Using NetworkX.

